# Preparedness of Undergraduate Medical Students to Combat COVID-19: A Tertiary Care Experience on the Effectiveness and Efficiency of a Training Program and Future Prospects

**DOI:** 10.7759/cureus.22971

**Published:** 2022-03-08

**Authors:** Poorvi Kulshreshtha, Yogesh Bahurupi, Mridul Dhar, Sameer Sharma, Rajesh Kathrotia, Shalinee Rao, Manisha Naithani, Manoj Gupta

**Affiliations:** 1 Physiology, All India Institute of Medical Sciences, Rishikesh, Rishikesh, IND; 2 Community and Family Medicine, All India Institute of Medical Sciences, Rishikesh, Rishikesh, IND; 3 Anaesthesiology, All India Institute of Medical Sciences, Rishikesh, Rishikesh, IND; 4 Anesthesiology, All India Institute of Medical Sciences, Rishikesh, Rishikesh, IND; 5 Physiology, All India Institute of Medical Sciences, Rajkot, Rajkot, IND; 6 Pathology/Advanced Center of Continuous Professional Development, All India Institute of Medical Sciences, Rishikesh, Rishikesh, IND; 7 Biochemistry/Advanced Center of Continuous Professional Development, All India Institute of Medical Sciences, Rishikesh, Rishikesh, IND; 8 Radiation Oncology, All India Institute of Medical Sciences, Rishikesh, Rishikesh, IND

**Keywords:** electrocardiography, personal protective equipment, covid-19, medical students, training programs

## Abstract

Introduction

Due to the nature of the coronavirus disease 2019 (COVID-19) pandemic, final year medical undergraduate students have had to be involved in patient management in different countries. The same was the case with India. This study was conducted with the objective to analyze the effectiveness and efficiency of preparedness training to combat COVID-19 in pre-final and final-year medical students at a tertiary care institute in North India.

Methods

A pre-post study was conducted among final and pre-final year medical undergraduate students. Data was collected as pre-test and post-test multiple-choice questions (MCQs) and clinical vignettes.

Results

A total of 179 medical undergraduate students attended the training. Scores on general instructions, personal protective equipment (PPE) donning and doffing, hand hygiene, biomedical waste management, contact tracing, cleaning and disinfection, ECG, and COVID-19 management improved significantly after the training. Pre-test scores on ECG, simulation, COVID-19 management were 21.58±5.311, 17.05±4.501, and 23.84±4.067, respectively. Post-test scores on ECG, simulation, COVID-19 management were 28.01±6.826, 23.84±4.067, and 6.93±1.726, respectively. Pre-test and post-test scores were statistically significant (p=0.0001).

Discussion

Our preparedness training program was effective in delivering the intended skills. The efficiency of the training program was demonstrated through simulation. We created a trained pool of medical undergraduate students to assist clinicians in COVID-19-related supportive care.

## Introduction

Acute surges in coronavirus disease 2019 (COVID-19) cases have witnessed a massive trained medical manpower crunch across the world, and the magnitude of COVID-19 infections has overarched existing health infrastructure. Various measures have been taken to contain the manpower crisis, including recruiting medical personnel from non-COVID-19 areas, cancelling the leaves of hospital staff, and rehiring retired doctors and nursing staff [1]. However, COVID-19 patients have overwhelmed healthcare facilities, leading to insufficient beds as well as health professionals.

This unprecedented crisis urged taking bold steps to rapidly increase human resources in the healthcare sector. One way of responding to the pandemic was to induce and train resident doctors to become community health workers [1]. The government of Ireland requested “healthcare professionals from all disciplines, not in the public health service, to register to be” an on-call COVID-19 service [2]. Some countries like Kenya also invigorated the recruitment drive for additional doctors in public hospitals [1]. Depending on their skill set, medical students were being considered for induction in the NHS to help with the response to COVID-19, with the scope of work ranging from administration to physician assistants. It is possible that the final year medical students who are willing to work can be accommodated [3]. 

A review of the literature documents the responsibilities that medical students can assume, including clinical history taking, communicating results of the COVID-19 test to individuals, educating the patient on COVID-19 care, documenting visits, and fielding questions about COVID-19 [4]. 

The government of India issued an advisory to address and augment human resources to manage the COVID-19 situation. One of the emphases given in the advisory was the utilization of the “Final Year Bachelor of Medicine and Bachelor of Surgery (MBBS) students for providing services such as teleconsultation and monitoring of mild COVID-19 cases after due orientation by and under the supervision of the faculty” [5].

In a Polish study, it was observed that volunteering by students earns respect and gratitude and grants professional experience, a sense of giving real aid, and the development of collaboration skills. However, the same study raised concerns about students’ limited knowledge, which could be the main hindering factor for volunteering and hesitation to take responsibility. Therefore, students should be sufficiently educated, and information should be provided explicitly for the successful delivery of services. Updated information can also be shared with students through online videos and handouts [6].

As per the need assessment and directives of the Health Ministry of India at our institute, preparedness training of pre-final and final-year medical students was conceived, planned, and implemented to expand the manpower pool to support clinicians in COVID-19-related patient care and utilize their services as and when required. 

This study was conducted to analyze the effectiveness and efficiency of preparedness training to combat COVID-19 in pre-final and final-year medical students. In addition, this study also aimed to delineate the challenges and identify the facilitating and hindering factors faced during training. 

## Materials and methods

A pre-post study (educational interventional study) was conducted on final and pre-final year students in the Advanced Center of Continuous Professional Development (CPD) at a tertiary care institute in Uttarakhand, India. The Institute Ethics Committee of All India Institute of Medical Sciences, Rishikesh, India, approved the study vide letter no. AIIMS/IEC/21/336. Pre-final year and final year students undergo regular clinical postings as part of their curriculum. The training program for COVID-19 was a well-planned and interactive course intended to enhance the cognitive and psychomotor skills of the students. Various modules were designed to allow students to attain the desired competencies. The content was prepared and validated by experts, and training was delivered by faculty and tutors from medical and nursing specialities over a two-day period (Table 1). A session on the principles of ECG recording, identification of abnormal rhythms, and clinical management of some common arrhythmias in COVID-19 patients were delivered through an interactive lecture. A session on COVID-19 management protocols and treatment algorithms was discussed as a large group session. Training on general instructions, personal protective equipment (PPE) donning and doffing, hand hygiene, biomedical waste management, contact tracing, cleaning, and disinfection was provided using video clippings. Skill-based learning was performed on the second day of training using a simulation facility on medium-and low-fidelity simulators (Table 1). Information on the duration of the session, instructors were added as a supplementary file.

**Table 1:** Training modules and number of participants trained PPE: personal protective equipment; ABG: arterial blood gas analysis, CPR: cardiopulmonary resuscitation, ECG: Electrocardiogram; MBBS: Bachelor of Medicine Bachelor of Surgery S. No 1, 2 and 3: Day 1 of training S. No 4: Day 2 training

S. No	Name of Training	Designation	Number of Participants
1	Training on general instructions, PPE donning & doffing, Hand hygiene, biomedical waste management, contact tracing, and cleaning and disinfection	MBBS Final Year Students	97
		MBBS Pre-Final Year Students	88
Total			185
2	Training on ECG identifications of rhythm and clinical management in COVID-19 cases	MBBS Final Year Students	93
		MBBS Pre-Final Year Students	95
Total			188
3	COVID-19 management protocols and treatment algorithms	MBBS Final Year Students	97
		MBBS Pre-Final Year Students	91
Total			188
4	Simulation-based training on ECG, airway management and adjuvants, bag and mask (contraindicated in COVID-19), vital monitoring and ABG, compression-only CPR, proning procedure	MBBS Final Year Students	96
		MBBS Pre-Final Year Students	89
Total			185

A pre-test session and a post-test session were conducted through multiple-choice questions (MCQs) and clinical vignettes to evaluate the effectiveness of the training program. Efficiency was assessed through skill-based learning as an objective structured clinical examination (OSCE) using a pre-validated checklist and MCQs. A cut-off score of 70% was considered for the successful performance of the skill. A cut-off score of 70% was considered for successful completion of the written test using the MCQ mode. The number of participants trained in two days and the number of instructors who facilitated the training were evaluated to assess the efficiency of the training program. Feedback on facilitating and hindering factors and each session of the training program was obtained from the participants.

Statistical analysis

Individual pre-test and post-test scores were entered into Microsoft Excel (Microsoft Corp., Redmond, Washington, United States). The mean pre-test and post-test scores were calculated and compared using the paired t-test for statistical significance. IBM SPSS Statistics for Windows, Version 23.0 (Released 2015. IBM Corp., Armonk, New York, United States) was used for statistical analysis. Feedback on the training program was compiled in percentages and proportions. Responses of open-ended questions were categorised into appropriate categories. Statistical significance was set at p < 0.05.

## Results

A total of 179 MBBS students participated in COVID-19 training over two days. Of these, 153 completed ECG training, 157 completed general instruction training, 179 completed simulation training, and 169 completed COVID-19 management training under the guidance of instructors.

Test scores for general instructions, PPE donning and doffing, hand hygiene, biomedical waste management, contact tracing, cleaning and disinfection, ECG, and COVID-19 management improved significantly after training (p=0.0001) (Table 2). On the assessment of participants for skill training using OSCE for six different skills, all participants scored more than the essential cut-off of 70%. Average scores for cardiac monitoring, prone positioning, compression-only cardiopulmonary resuscitation (CPR), airway management, ECG, and arterial blood gas analysis (ABG) were 88%, 87%, 94%, 93%, 94%, and 94%, respectively.

**Table 2:** Mean pre-test and post-test scores of participating students (values are Mean±SD) PPE: personal protective equipment; COVID-19: coronavirus disease 2019

Training module	Pre-test score	Post-test score	P value
ECG (N=153)	21.58±5.311	28.01±6.826	0.0001
General Instruction, PPE donning and doffing, hand hygiene, biomedical waste management, contact tracing and cleaning and disinfection (N=157)	22.05±3.844	26.31±4.912	0.0001
Simulation (N=179)	17.05±4.501	23.84±4.067	0.0001
COVID-19 management (N=169)	6.04±1.652	6.93±1.726	0.0001

Feedback on COVID-19 training

In our study, 161 (97%) students were satisfied with the training and felt that the module was of a high standard. About 158 (94.7%) students wrote that the teaching staff was enthusiastic, and the education delivered was relevant to learning outcomes. About 95% of the students felt that the module was intellectually stimulating, and 91% responded that the module was relevant to practice. Support and guidance were available to 92% of the students, and 91% answered that they received constructive feedback. A total of 92% expressed that the learning resource material was of a high standard, and 93% responded that hands-on teaching enhanced their learning (Table 3).

**Table 3:** Feedback of MBBS students on COVID-19 hands-on training (N=167) MBBS: Bachelor of Medicine Bachelor of Surgery; COVID-19: coronavirus disease 2019

Sr No	Feedback	Strongly disagree	Disagree	Neutral	Agree	Strongly agree
		N (%)	N (%)	N (%)	N (%)	N (%)
1	Overall, I was satisfied with the quality of the module	1 (0.60%)	1 (0.60%)	3 (1.80%)	59 (35.30%)	103 (61.70%)
2	Teaching on the module was of a high standard	1 (0.60%)	3 (1.80%)	7 (4.20%)	62 (37.10%)	94 (56.30%)
3	Teaching staff were enthusiastic about the module	1 (0.60%)	1 (0.60%)	7 (4.20%)	37 (22.20%)	121 (72.50%)
4	The material taught was relevant to the module Learning Outcomes	1 (0.60%)	1 (0.60%)	4 (2.40%)	51 (30.50%)	110 (65.90%)
5	The module content was intellectually stimulating	1 (0.60%)	1 (0.60%)	13 (7.80%)	43 (25.70%)	109 (65.30%)
6	The content of the module was informed by relevant research and/ or practice	0	3 (1.80%)	10 (6.00%)	54 (32.30%)	100 (59.90%)
7	Sufficient support and guidance was available from the teaching staff when needed in the module	1 (0.60%)	2 (1.20%)	8 (4.80%)	48 (28.70%)	108 (64.70%)
8	I received constructive feedback on my work during the module which helped me assess my progress	0	2 (1.20%)	12 (7.20%)	51 (30.50%)	102 (61.10%)
9	Learning materials and resources were of a high standard and supported my learning well	1 (0.60%)	4 (2.40%)	8 (4.80%)	53 (31.70%)	101 (60.50%)
10	The hands-on teaching enhanced my learning on the module	2 (1.20%)	2 (1.20%)	10 (6.00%)	44 (26.30%)	109 (65.30%)
		Very poor	Poor	Average	Good	Excellent
11	Content of program	0	0	5 (3.00%)	64 (38.30%)	98 (58.70%)
12	Delivery of concerned content	0	1 (0.60%)	5 (3.00%)	66 (39.50%)	95 (56.90%)
13	Trainee and resource faculty Integration	0	0	4 (2.40%)	67 (40.10%)	96 (57.50%)
14	Time accorded for training	Inadequate			Adequate	
		18 (10.80%)			149 (89.20%)	
15	Method used for training	Inappropriate			Appropriate	
		6 (3.60%)			161 (96.40%)	
		No			Yes	
16	Enhancement of skills after training	2 (1.20%)			165 (98.80%)	
17	Usefulness of training content	1(0.60%)			166 (99.40%)	

The time required for training was adequate, as per 89% of respondents, and the methods used were appropriate, as expressed by 96%. Of the participants, 165 (98.8%) felt that their skills were enhanced while 166 (99.4%) felt that the training content was useful (Table 3). The most helpful sessions mentioned by the majority (111 (66 %)) were CPR and ABG followed by ECG, intubation, and cardiac monitoring (Table 4).

**Table 4:** Feedback on most useful session, sessions requiring elaboration and new inclusions ATLS: advanced trauma life support; CPR: cardiopulmonary resuscitation; ABG: arterial blood gas analysis

Most useful sessions	Sessions need elaboration	Other topics to be included
Compression-only CPR and ABG: 111 (66.5%)	Cardiac monitoring: 56 (33.5%)	Emergency management: 58 (34.7%)
ECG: 7 (4.2%)	Airway: 38 (22.8%)	ATLS: 7 (4.2%)
Intubation: 6 (3.6%)	Compression-only CPR: 15 (9.0%)	Monitoring: 2 (1.2%)
Cardiac monitoring: 5 (3.0%)	Ventilator: 6 (3.6%)	Radiography analysis: 2 (1.2%)
Compression-only CPR: 5 (3.0%)	ABG: 9 (5.4%)	On real person: 1 (0.6%)
Prone positioning: 3 (1.8%)	Intubation procedure: 7 (4.2%)	-
Ventilator: 1 (0.6%)	Proning: 1 (0.6%)	-

Interactive sessions and hands-on experience were the main facilitatory factors highlighted by 36% and 24% of participants, respectively. About 19% of participants reported time as a limiting factor, and 14% of students listed the length of some session being too long as a hindering factor (Table 5).

**Table 5:** Facilitatory and hindering factors during training

Facilitatory Factors	Hindering Factors
Interactive session: 61 (36.5%)	Time limit: 33 (19.8%)
Hands on experience: 41( 24.6%)	Long session: 24 (14.4%)
Everything: 22 (13.2%)	Lack of real patient: 6 (3.6%)
Language: 1 (0.6%)	Lack of adequate space: 13 (7.8%)
-	The temperature in training hall: 7 (4.2%)
-	Language: 1 (0.6%)
-	Network issues: 1 (0.6%)

## Discussion

A structured program was planned and executed to ensure preparedness for final and pre-final year medical students to overcome the demand for supportive staffing with clinicians to combat COVID-19. In this context, they had to be trained adequately before they could be put into clinical duties [7]. It has been realized that senior medical students have sufficient clinical acumen and practical erudition. They possess months of regular training in skills such as identification, recognition, reasoning, and decision-making, and work under a huge workload for longer hours. They are also exposed in their pre- and para-clinical years to the importance of soft skills and ethical issues, which helps to investigate the epidemiological characteristics and symptomology [8].

The course material for COVID-19 preparedness training was mostly aligned with Harden’s principles on the integration ladder as a tool for planning, implementing, and evaluating the effectiveness of course content [9]. All steps of the ladder viz isolation, awareness, harmonization, nesting, temporal coordination, sharing, correlation, complementary, multidisciplinary, interdisciplinary, and transdisciplinary were included during the design and execution of the course [9]. Medical and nursing faculty members delivered the course content, and some of the sessions witnessed the active participation of resident doctors and nursing officers in addressing education pertaining to all domains [10]. There was a good improvement in knowledge among participants following the training session. 

Intense brainstorming is essential when designing the course content and its implementation phases to effectively deliver the appropriate content. The purpose of our training program was to increase the core competencies required for COVID-19 preparedness among final and pre-final year medical students. This program was a unique learning opportunity for budding medical personnel to prepare them with the desired competencies to respond to challenges during the COVID-19 pandemic. 

Medical students are an essential workforce, and experience shows that their recruitment as volunteers in various capacities, such as gathering patient feedback, has shown promising results. Ample opportunities exist for the students to get real-time, hands-on training of the teamwork and coordination required and pressures faced by senior functionaries in a hospital. They also get a glimpse of the functioning and coordination of various areas in a hospital, how the wards function, and how the healthcare team works as a unit to provide care [11].

A study conducted to compare attitudes and perceived outcomes of students who volunteered may help shape volunteering infrastructure during future pandemics and identify the existing structure of the current clinical scenario. Such findings may help to develop and re-structure medical electives, clinical placements, and apprenticeships. Future longitudinal research may address the effects of volunteering on academic performance and career progression [12]. A study conducted in Indonesia explored the state of preparation and perceptions of volunteers during this pandemic. Knowledge and understanding of COVID-19 pathophysiology were also been studied. A questionnaire to address professional identity formation and determine readiness was utilized, which included adaptations and coping strategies, perceived roles during the pandemic COVID-19 management, and any previous similar experience to volunteer. It was found that male gender, public university students, and low family income were the key indicators for voluntary engagement with the community and hospital [13].

The present COVID-19 pandemic has unraveled our inherent unpreparedness and deficient infrastructure to combat any such crisis. Deployment of students in the hospitals in the present calamity also reinforces and inculcates important morals and standards such as altruism, service, and professional solidarity and integrity [4]. Since the pandemic is ongoing, the approach used in Vietnam might be helpful for other resource-scarce settings in conducting active and prompt responses during the pandemic. Initiation of training courses on the essential medical understanding of COVID-19, about 97 senior students in preventive medicine and 27 final-year students of public health were recruited to support prevention, early detection, and control of COVID-19, along with the health staff at the Centers for Disease Control (CDC), Vietnam. The students also volunteered to conduct an epidemiological investigation of cases from epidemic areas. They supported the health system by offering telephonic counseling, collecting samples from suspected people, data entry, and disinfection for COVID-19 [8].

Final-year medical students’ services were advised worldwide in light of acute workforce crises during the COVID-19 pandemic [14]. In our study, the majority of the students were satisfied with the training. The knowledge and enhancement of skills gained during the session can be applied in the future in the workplace. The majority responded that the time required for training was sufficient, and the method used for training was appropriate in the setting of the COVID-19 pandemic. Interactive sessions were critical facilitatory factors in the training program.

Deployment of qualified and willing medical students was introduced in many countries, such as Canada, Ireland, and Italy, during the worst phases of the COVID-19 pandemic in the face of an unprecedented shortage of qualified healthcare workers. To expedite the recruitment of students, practices such as preponing final year exams were undertaken in some American medical schools. Students were adequately provided with sufficient PPE, adequate testing, and were allowed only limited contact with patients [15]. Premorbid students participated in patient care via telehealth or other noncontact duties, such as research and running call centers, to provide guidance. Students’ activities ranged from developing educational materials for the general public, childcare services, and grocery delivery to donations of PPE to distribute to hospitals and community organizations [15].

Volunteering for clinical duties could be one way to overcome the shortcomings and disruptions caused to medical education by the ongoing COVID-19 pandemic. The concept of service-learning, wherein novices can learn by working under supervision and graduate to become proficient and gain expertise has gained momentum over the past few years [16].

Such service-based roles with a clinical team should continue even if the pandemic is over to ensure continuity of lessons learned about deficiencies regarding the current medical curriculum within medical education. The traditional postings in the clinical areas are aimed at extracting maximum information from patients about illnesses and corroborating that information with what they learn in theory only. However, the service roles encourage volunteers to build confidence and management skills that could foster their personal growth and help raise a workforce that could be handy in times of pandemics. Our COVID-19 preparedness program was explicitly designed to combat any future waves of COVID-19, as well as other potential disasters. The inclusion of service-based learning early in curricula may help develop a community-based perspective for clinical practice [17].

Such an integrated framework encompassing all Bloom’s taxonomy domains also lays the foundation for COVID-19 preparedness, as delivered in our training program. Such designs have been utilized in the influenza epidemic in China, and experience can be adapted to various public health environments with need-based modifications and implemented in multiple healthcare settings [18]. In a study conducted in the United Kingdom (UK) to identify the impact of COVID-19 on final year medical students’ examinations and placements in the UK, some students felt that assisting in hospitals during the pandemic would be fruitful and enhance learning. There is an unprecedented opportunity to integrate and learn by shadowing doctors [19].

It is essential to understand that events such as pandemics and large-scale natural disasters may disrupt medical education on the one hand. On the other hand, these challenges serve as an initiating point for brainstorming to bring about educational restructuring. A paradigm shift in the format of the medical curriculum is necessary, which should also include pandemic preparedness to make students equipped during a crisis. Such preparedness programs will reap further benefits in their careers [20]. A study on disaster preparedness in a medical school showed that 70% of students felt unprepared to participate in an emergency, and 11% of students rated themselves as unprepared after training. In light of the possibility of more pandemics in the future, all prospective health personnel should be adequately trained. Inclusion of need-based specific content in the current preparedness content assures academic competency and brings logistical challenges to the forefront [20].

 Limitations

Despite the results showing improvement in all domains covered in the COVID-19 preparedness program, some limitations existed in our study. The real impact of training can be evaluated better by students’ performance during job roles.

## Conclusions

Our preparedness training program effectively and efficiently delivered knowledge and skills to pre-final and final year medical students. Good workforce strength was created in a short time span to assist clinicians in COVID-19-related supportive care. Curriculum revision to include basic skill development on critical care and disaster management as mandatory skill assets for medical graduates should be undertaken. A healthy culture of volunteering in community programs should be encouraged and explicitly included in the curriculum to foster a service mindset among medical graduates. Policies and protocols should be laid down by statutory bodies for epidemic/pandemic responses that specify medical students’ roles and responsibilities. 

## Appendices

**Table 6:** Pre-test and post-test tool consisted of psychomotor, cognitive, and affective domains COVID-19: coronavirus disease 2019

Psychomotor skill domain (6 items)	Cognitive domain (3 headings with 91 items)	Affective domain (25 items)	Overall total score
Minimum score: 0	ECG: 45 items	Minimum score: 0	Minimum score: 0
Maximum score: 92	General instructions: 36 items	Maximum score: 25	Maximum score: 208
-	COVID-19 management: 10 items	-	-
-	Minimum score: 0	-	-
-	Maximum score-91	-	-

**Table 7:** Details of various instructor-facilitated training programs PPE: personal protective equipment; CPR: cardiopulmonary resuscitation; ABG: arterial blood gas; COVID-9: coronavirus disease 2019 A two-day training program was conducted separately for the final year and the prefinal year. The hands-on session was conducted in small groups in the simulation laboratory.

S No	Name of Training	Number of instructors	Duration
1	Training on general instructions, PPE donning & doffing, hand hygiene, biomedical waste management	02	3 hours
2	Training on ECG identifications of rhythm & clinical management in COVID-19 cases	02	3 hours
3	Simulation based (in-person hands-on training) ECG, bag & mask (contraindicated in COVID-19), airway adjuvant, vital monitoring & ABG, compression-only CPR, proning procedure	08	8 hours
4	COVID-19 management protocols & treatment algorithms	01	3 hours
